# Chronic Hypoxia Due to Hypopharyngeal Cancer Presenting As Generalized Weakness: A Case Report

**DOI:** 10.7759/cureus.86563

**Published:** 2025-06-22

**Authors:** Ryunosuke Hashikawa, Akira Kuriyama, Takumi Terai, Keigo Honda

**Affiliations:** 1 Department of Primary Care and Emergency Medicine, Kyoto University Graduate School of Medicine, Kyoto, JPN; 2 Department of Head and Neck Oncology and Innovative Treatment, Kyoto University Graduate School of Medicine, Kyoto, JPN

**Keywords:** airway, generalized weakness, hypopharyngeal cancer, hypoxia, stridor

## Abstract

Generalized weakness is a presentation well associated with various underlying medical conditions such as infection and malignancy. However, the association between generalized weakness and hypoxia has rarely been described. A female patient in her late 80s with generalized weakness was admitted to our emergency department. Inspiratory stridor and hypoxia were incidentally recognized, leading to the diagnosis of hypopharyngeal cancer (HPC) with chronic hypoxia. After oxygen supplementation, the generalized weakness completely resolved until the HPC started to occlude the airway. Her unusual presentation could indicate that she did not compensate for her deteriorating hypoxia due to progressive obstruction of her upper airway. This case highlights the importance of a thorough physical examination in diagnosing generalized weakness, empowering physicians to closely evaluate the cause of generalized weakness and recognize it as a potential presentation of chronic hypoxia.

## Introduction

Weakness is a common presentation in the emergency department (ED), with up to 10% of ED patients presenting with this complaint [1]. Especially, generalized weakness accounts for 65% of the complaints, often necessitating comprehensive history taking and physical examinations due to the multitude of differential diagnoses [2]. Typical causes include infection (32%) and metabolic abnormalities (18%) [1]. Proinflammatory cytokines from infections induce central nervous system-mediated nonspecific symptoms, including weakness, lethargy, or listlessness [3]. Volume depletion, with or without hypotension, due to infection or metabolic abnormalities can cause global and/or tissue hypoperfusion, resulting in generalized weakness [4]. Electrolyte disturbances, such as hypokalemia and hypermagnesemia, can also induce generalized weakness by inhibiting neuronal depolarization and impairing muscle contraction [5]. However, an association between generalized weakness and hypoxia has rarely been reported. We encountered a case of generalized weakness. Her unexpectedly detected inspiratory stridor led to the diagnosis of hypopharyngeal cancer (HPC).

## Case presentation

An 86-year-old woman presented to our ED with generalized weakness. She was transferred to our ED after developing weakness in her extremities. She had been healthy and could ambulate without claudication prior to the visit. She denied experiencing shivering chills, upper respiratory symptoms, dyspnea, or weight loss. She denied having any pain in her chest, abdomen, or back. She denied any triggers for her symptoms, such as aspiration or a recent history of burns. She had no known allergies to food or drugs, and she denied ingesting anything immediately before the presentation. Her medical history included hypertension, dyslipidemia, and cerebral infarction with minimal right-sided coordination deficits. She had never smoked or consumed alcohol. Upon admission to the ED, her vital signs were as follows: blood pressure of 132/89 mmHg, respiratory rate of 24/min with an oxygen saturation of 88% on room air, temperature of 36.7℃, and pulse of 108/min and regular. Chest auscultation showed no abnormal cardiac murmurs or pulmonary sounds; however, an unexpectedly strong inspiratory stridor in her neck was present. She had no skin changes, such as wheals or facial burns. She had complete manual muscle testing (MMT) scores for all extremities.

**Figure 1:**
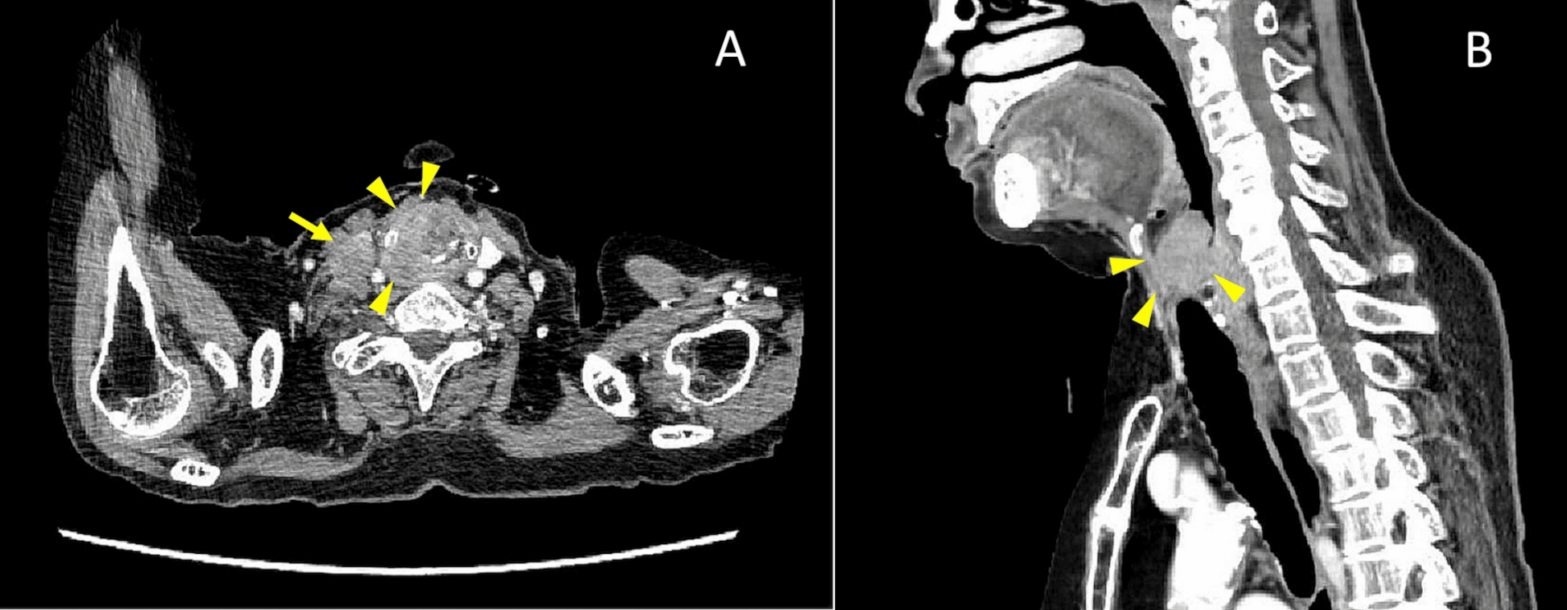
Neck contract-enhanced computed tomography (A: axial; B: sagittal) This reveals a hypopharyngeal mass (arrowheads) with invasion of the thyroid cartilage and enlargement of the right neck lymph node (arrow).

Arterial blood gas analysis on the ambient air revealed hypoxia. Her hemoglobin concentration, leukocyte count, and electrolytes, including potassium, calcium, and magnesium, were insignificant (Table 1). Contrast-enhanced computed tomography and magnetic resonance imaging revealed a pharyngeal mass obstructing the upper airway tract, with thyroid cartilage invasion and right neck lymph node enlargement without apparent pulmonary lesions (Figure 1).

**Table 1:** Laboratory test results upon the emergency department visit

Parameter	Unit	Patient	Reference range
Complete blood counts			
Hemoglobin	(g/dL)	13.5	11.5-16
Hematocrit	(%)	39.7	31.7-48
Platelet count	(×10^3^/mm^3^)	296	175-300
White blood cell	(×10^3^/mm^3^)	8.6	4-9
Neutrophil	(%)	60.1	
Lymphocyte	(%)	33.3	
Monocyte	(%)	6.2	
Blood metabolic panel			
Sodium	(mmol/L)	142	136-145
Potassium	(mmol/L)	3.8	3.5-5.1
Chloride	(mmol/L)	103	98-107
Glucose	(mg/dL)	132	70-99
Blood urea nitrogen	(mg/dL)	16	6-24
Total protein	(g/dL)	7.2	6.0-8.5
Albumin	(g/dL)	3.5	4.0-5.0
Alkaline phosphatase	(U/L)	86	39-117
Aspartate aminotransferase	(U/L)	24	0-40
Alanine aminotransferase	(U/L)	17	0-40
Calcium	(mg/dL)	9.1	8.7-10.2
Magnesium	(mg/dL)	1.7	1.5-3.0
Total bilirubin	(mg/dL)	0.6	0-1.2
Procalcitonin	(ng/ml)	0.05	0-0.1
Arterial blood gas			
pH		7.45	7.35-7.45
PaCO2	(mmHg)	38.5	35-45
PaO2	(mmHg)	63.9	80-100
HCO3-	(mmol/L)	27	22-27

**Figure 2:**
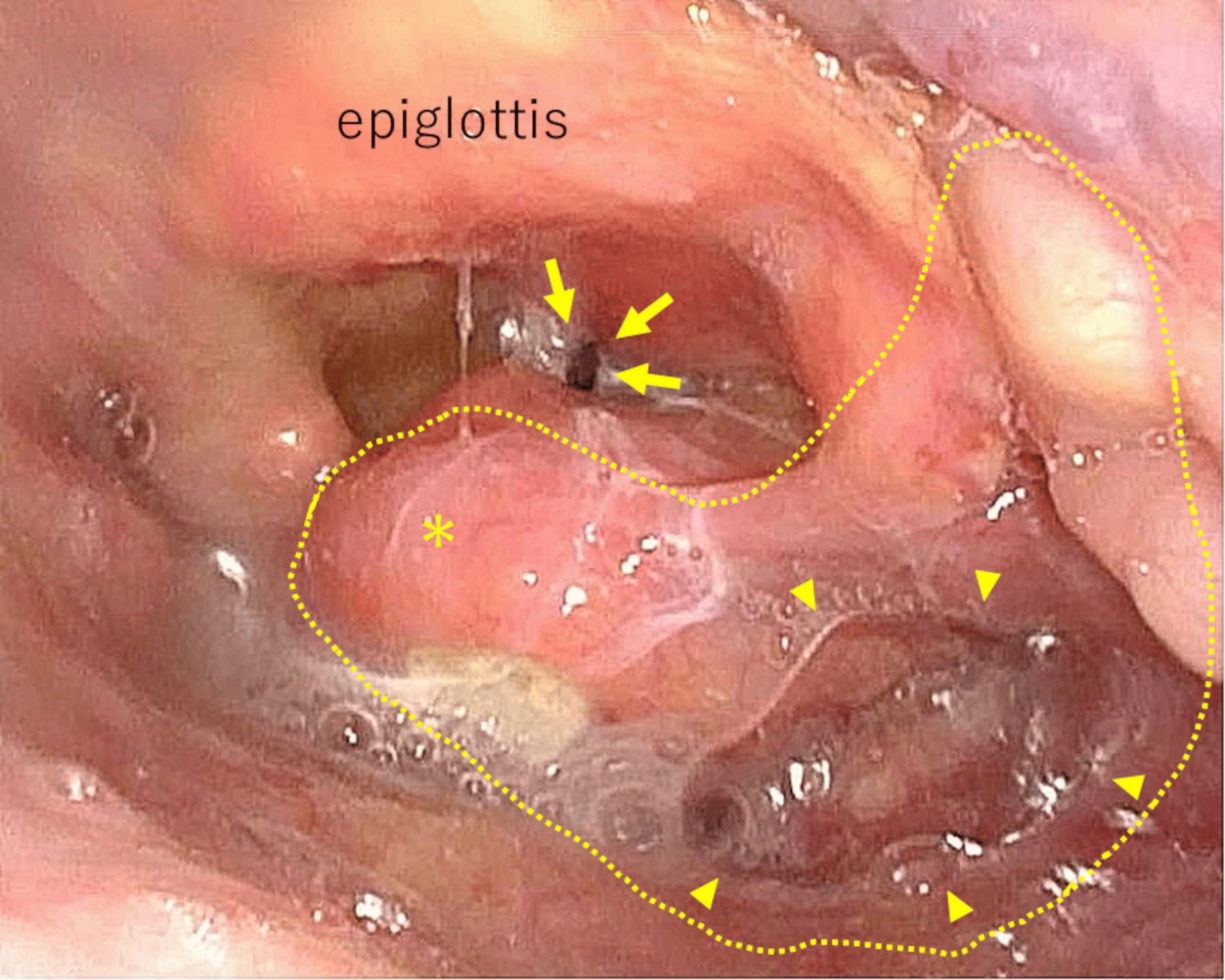
Flexible laryngoscopy It revealed the presence of a mass (dotted line) with mucosal ulcerations (arrowheads) extending underneath the mucosa (asterisk) on the right pyriform sinus, nearly obstructing the glottis (arrows).

**Figure 3:**
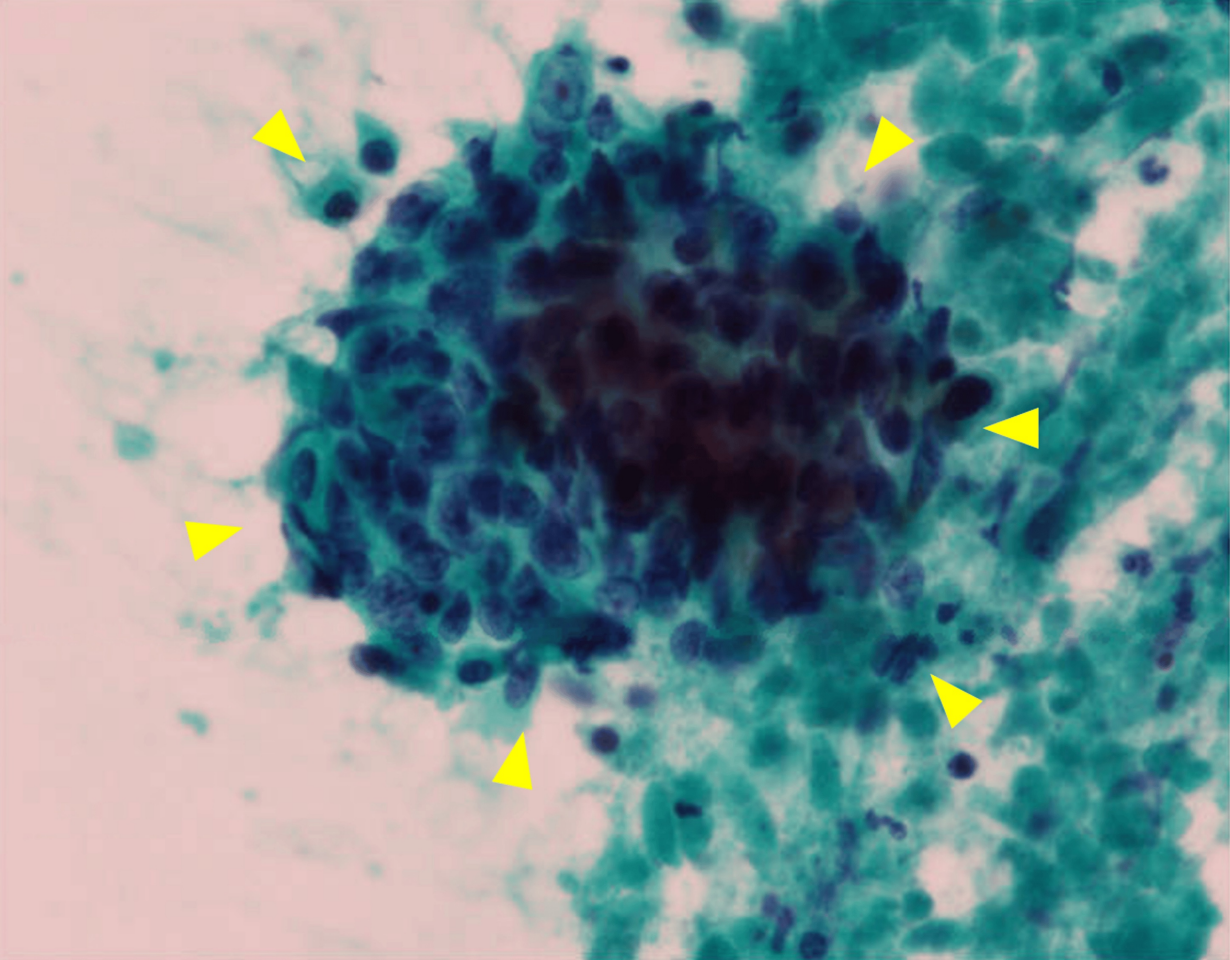
Micrograph of fine needle biopsy of lymph node The cells showed epithelial differentiations with various shapes and increased nuclear-to-cytoplasm ratio (arrowheads), which highly indicated squamous cell metastasis.

Flexible laryngoscopy revealed a mass with mucosal ulcerations in the right pyriform sinus (Figure 2). A needle biopsy of the enlarged lymph node of the neck showed variably sized nests of squamous cells with an increased nuclear-to-cytoplasm ratio, suggesting squamous cell carcinoma metastasis (Figure 3). These findings confirmed the diagnosis of HPC with thyroid cartilage invasion and lymph node metastasis.

After admission, the patient was able to walk without fatigue with 2 L/min of oxygen. However, as her HPC gradually expanded over time, dyspnea on exertion progressively worsened 15 days after admission. Although there was a high risk of complete airway obstruction by the tumor, the patient refused any invasive treatment, including tracheostomy. One month after admission, the patient was transferred to another hospital for long-term oxygen therapy.

## Discussion

HPC is a relatively uncommon cancer; approximately 2,500 new cases are annually diagnosed in the United States [6]. It typically develops in males older than 50, and known risk factors are excessive alcohol and tobacco use [7]. This cancer has poor survival because the hypopharynx is rich in highly anastomotic regional lymphatics, leading to early dissemination of the tumor [8,9]. The tumor is often large enough to cause local pressure on nearby structures or obstruct the upper airway by the time symptoms are reported [10,11]. Common presentations of HPC are neck mass, sore throat, hoarseness, and dysphagia, which are often misconceived as minor or insignificant symptoms [12,13]. To our knowledge, extremity weakness has not been reported as the initial symptom of HPC.

There are numerous differential diagnoses for generalized weakness. A prospective study of 935 non-trauma patients with weakness found that infection, followed by metabolic dysfunctions and malignancies, was the most common cause of generalized weakness [1]. Patients with infections or malignancies experience generalized weakness, which is induced by proinflammatory cytokines acting on the central nervous system, such as interleukin-1 (IL-1) and tumor necrosis factor (TNF). Numerous studies show that cytokines injected into the body can cross the blood-brain barrier, impairing the ability to engage in social interactions or seek food, which is associated with symptoms of illness. These symptoms include reduced exploratory behavior and decreased food intake [3]. Dehydration leads to global tissue hypoperfusion and electrolyte disturbances interfere with neural activity and muscle contraction [5]; correction of these statuses could mitigate the weakness. However, in our patient, hypoxia with inspiratory stridor was incidentally observed. Her medical history and physical examinations did not indicate common causes of inspiratory stridor, such as anaphylaxis, foreign body aspiration, or airway burns. Ultimately, her unexpected inspiratory stridor led to the diagnosis of HPC as the underlying cause of transient generalized weakness.

Oxygen supplementation after admission led to her improved exercise tolerance and no recurrence of her generalized weakness. This supports that acute exacerbation of chronic hypoxia directly induces transient generalized weakness. The silent clinical course before admission suggested that hypoxia in our patient could have been chronic; dyspnea developed only after admission. We, therefore, speculate that she could not temporarily compensate for the progressively worsening hypoxia for the first time on the day of admission. Alternatively, the enlarging HPC could have temporarily obstructed her airway, which led to exacerbation of chronic hypoxia and subsequent, transient generalized weakness.

## Conclusions

Chronic hypoxia is difficult to observe. Our case highlights that acute exacerbation of chronic hypoxia could be another differential diagnosis of transient generalized weakness and highlights the importance of thorough physical examinations in diagnosing chronic hypoxia. Clinicians must closely investigate the cause of generalized weakness and recognize it as a potential presentation of chronic hypoxia due to diseases of the upper and lower airways, including HPC.
